# Prevalence and characteristics of frequent presenters to Auburn Hospital emergency department

**DOI:** 10.1111/1742-6723.14011

**Published:** 2022-05-18

**Authors:** Billie Cordell, Khanh Nguyen, Ahmed K Shahir, Sarah J Lord, Gisselle Gallego

**Affiliations:** ^1^ Auburn Clinical School, The School of Medicine The University of Notre Dame Australia Sydney New South Wales Australia; ^2^ Emergency Department Auburn Hospital Sydney New South Wales Australia; ^3^ Medicine Department Auburn Hospital Sydney New South Wales Australia; ^4^ National Health and Medical Research Council Clinical Trials Centre The University of Sydney Sydney New South Wales Australia

**Keywords:** Australia, emergency department, frequent attender, frequent presenter, medical record database

## Abstract

**Objectives:**

Frequent presenters (FPs) to the ED are common and contribute to ED overcrowding. Our aim was to identify the proportion of FPs over a 12‐month period and to investigate the sociodemographic, clinical and attendance characteristics of FPs.

**Methods:**

A retrospective cohort study of adult patients (≥18 years) presenting to Auburn Hospital ED between 1 January 2018 to 31 December 2018. Patients with ≥4 presentations in 12 months were classified as FP. Multivariable logistic regression was used to assess associations between sociodemographic characteristics and FP.

**Results:**

During the study period, there were 22 679 presentations to the ED from 16 624 adult patients. FPs represented 5.1% (95% confidence interval [CI] 4.8–5.5) of the total population, but 15.8% of the total ED visits. Median age of FPs was 46 years (interquartile range 29–72), 51.9% were males. Age over 65 was the strongest determinant of FP (odds ratio [OR] 2.33; 95% CI 2.01–2.72 adjusted for sex). FP was more likely for Arabic speakers compared to English speakers (OR 1.54; 95% CI 1.28–1.86 adjusted for age and sex) and least likely for Mandarin speakers (adjusted OR 0.40; 95% CI 0.27–0.59).

**Conclusions:**

FPs represent a significant proportion of ED visits, yet a small proportion of ED patients. Our findings suggest that identifying ways to provide targeted services to older FPs may reduce the overall rates. The differences between language groups and FP highlights the importance of social context and culture when developing targeted interventions.

**Key findings**

- Older patients above the age of 65 were more commonly FPs than younger age groups. Within this older age group, those born outside Australia were more commonly FPs than Australian‐born, highlighting the importance of addressing the unique care needs of this patient group. In the younger age group, those born outside Australia were less likely to be FPs.
- We found different patterns of frequent ED use among different language groups. There was a strong association between Arabic speakers and FP, whereas Mandarin speakers were less likely to be FPs.
- These findings support the importance of further research to understand the reasons for these differences, as well as the specific needs of these cultural and linguistic groups in order to provide targeted interventions.

## Introduction

Worldwide, patients with a history of recurrent presentations to the ED within a specific period are a common occurrence.^1^, ^2^, ^3^ Often termed frequent presenters (FPs), these patients place a burden on healthcare system resources. Although FPs typically represent a small proportion of ED patients, they account for a disproportionate number of ED visits.^1^, ^2^, ^3^ They often have complex and long‐standing medical comorbidities and social needs^4^, ^5^ and rely heavily on other health services, including ambulance services, primary care and outpatient clinics.^6^ FPs also experience higher mortality than non‐FPs.^6^

Understanding the characteristics and determinants of FPs allows health service providers to address the specific needs of this population efficiently and cost‐effectively. Yet to date, most studies on FPs have been undertaken outside of Australia and may not be applicable to the Australian population and healthcare system.^7^, ^8^, ^9^, ^10^

One unanswered question is the relationship between specific groups within the culturally and linguistically diverse (CALD) population and frequency of presentation. CALD is defined as people born in non‐English speaking countries and/or who do not speak English at home.^11^ In Australia, the migrant population is growing rapidly, with over a quarter of the total population born overseas.^12^ Although Australia's multicultural groups are heterogeneous, there are common issues faced by CALD populations when interacting with the healthcare system, which contribute to poor health outcomes. As Phillips argues, the differences in health outcomes associated with different language groups have been attributed to factors relating to sociodemographic determinants, as well as difficulty accessing services, lack of health system responsiveness and language barriers.^13^

Given the evident need to expand the literature on FPs, the present study was undertaken to investigate the characteristics of FPs presenting to the ED of Auburn Hospital in Western Sydney. The aim was to identify the proportion of FPs over a 12‐month period and to investigate what sociodemographic, clinical and attendance characteristics are associated with frequent ED presentation.

## Methods

### Study setting

Western Sydney (WS) is one of the most CALD areas in New South Wales (NSW). Almost half of the population (47%) was born overseas and half (50%) speak a language other than English at home. India and China are the most frequent overseas countries of birth, whereas Arabic (13%) and Mandarin/Cantonese (18%) are the most frequent non‐English languages spoken at home.^14^ Auburn Hospital is a 155‐bed public hospital located in WS. It is classified as a major city acute group B level hospital in one of WS's Local Health District (WSLHD) and is a teaching hospital of the University of Notre Dame Australia (UNDA).

### Study design and cohort selection

This was a retrospective cohort study. The primary study cohort comprised all adult patients (18 years and older) who presented to Auburn Hospital ED between 1 January 2018 and 31 December 2018. For each patient, the ‘index’ presentation was defined as the first ED presentation recorded in the 2018 calendar year.

### Data collection

Data from all ED presentations between 1 January 2018 and 31 December 2018 were extracted from the Auburn Hospital ED electronic clinical record database. Study data included baseline patient characteristics at the index presentation (age, sex, country of birth, primary language, requirement of interpreter, marital status, health insurance status and Aboriginal and Torres Strait Islander status); clinical factors (triage category and presenting problem) and attendance factors (referral source to ED, transport to ED, date and time of attendance and discharge referral) at each presentation. Given prior evidence of the association between history of alcohol and drug abuse and history of mental illness and FP, we also extracted data for these conditions from the text recorded under ‘primary diagnosis’, by classifying descriptors that aligned with the ICD‐10‐AM codes F20–F69 to classify Mental Health disorders and ICD‐10‐AM F10–F19 to classify drug and alcohol‐related problems.

### Outcome measure

The primary outcome was frequency of presentation to the ED. The definition of FP is inconsistent across the literature.^10^ We therefore used the most common definition^1^, ^5^, ^10^ and defined ‘frequent’ as ≥4 presentations, and ‘very frequent’ as ≥10 presentations over a 12‐month period from the ‘index’ ED presentation date.

### Data analysis

We calculated the frequency of ED presentations for each subject over 12 months prospectively from the index presentation to identify ‘frequent’ and ‘very frequent’ presenters (vFP) within the subsequent 12‐month interval. We expressed the number of FP and vFP as a percentage of the study cohort with a 95% confidence interval (CI), and the total number of ED episodes recorded from subjects classified as FP and vFP as a proportion of all ED episodes recorded in 2018.

We described the sociodemographic and clinical characteristics of patients categorised by frequency of ED presentation (1–3 and 4+). To test associations between these characteristics and FP *versus* non‐FP, we used Pearson's *Χ* ^2^ test or Fisher's exact test (if cell frequencies <5) for categorical variables and Mann–Whitney's test for comparing the distribution of age in years. Patients with missing data for a characteristic were excluded from the analysis of that characteristic. We restricted our analysis of presenting problems to ‘common problems’; arbitrarily defined as problems recorded for ≥2% of the total population. In addition to the index visit, FPs who presented three or more times for a routine care visit were classified as having routine care as a presenting problem. Routine care was defined as care that could routinely be provided in a non‐emergency care setting, such as primary care (i.e. medicine administration, colostomy, catheter and plaster). We developed a multivariable logistic regression model to explore the independent effects of patient sociodemographic characteristics on frequency of presentation. We used the model to estimate an adjusted odds ratio for each variable examined. For this analysis, we adjusted age for sex; all other characteristics were adjusted for age in years (continuous variable) and sex. We included interaction terms to explore the potential interaction between two variables. Data were analysed using IBM SPSS Statistics for Windows (Version 26; SPSS Inc., Chicago, IL, USA).

The study was approved by the Human Research Ethics Committees of WSLHD (Reference (6158‐2019/STE16455) and UNDA (019163S).

## Results

**Figure 1:**
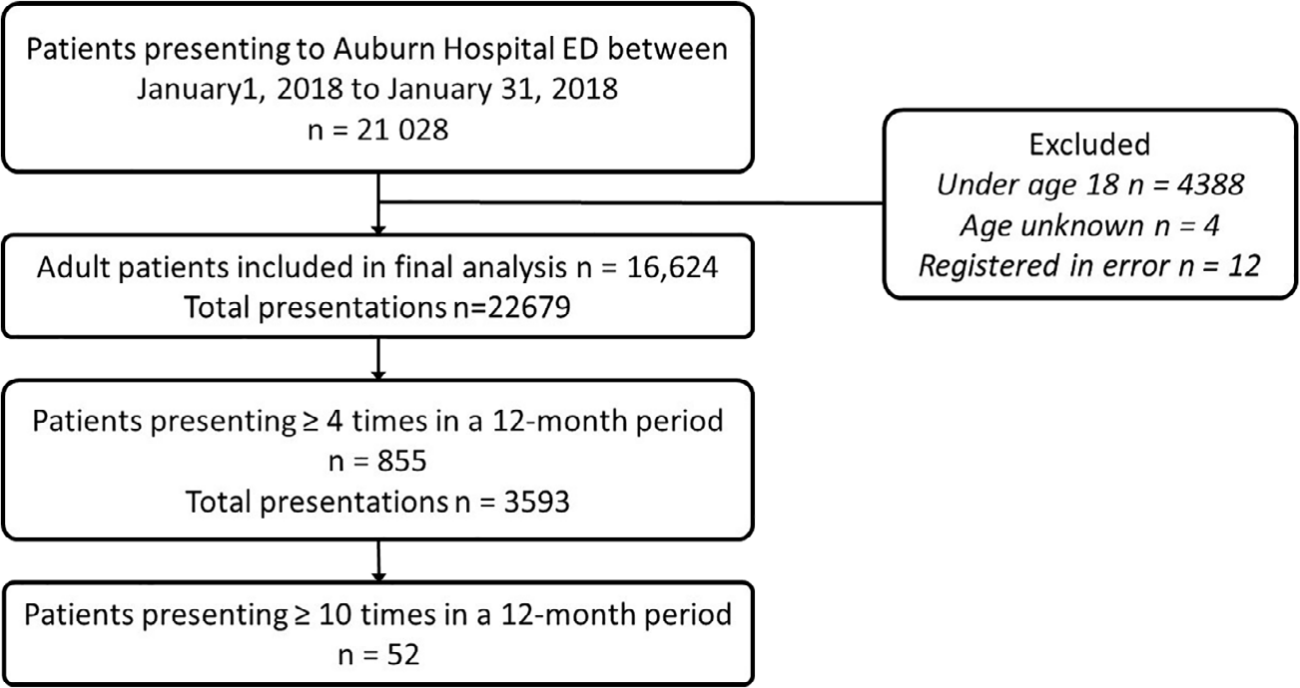
Flowchart of patients included in the study cohort.

During the period of 1 January to 31 December 2018, 16 624 adult patients presented to the Auburn Hospital ED a total of 22 679 times (Fig. 1). A total of 855 patients (5.1%, 95% CI 4.8–5.5) of all adult presenters in 2018 attended ED four or more times in 12 months from their first presentation in 2018 and were classified as FPs. This patient group presented 3593 times, which comprised 15.8% of the total ED episodes of care in 2018. Of the 855 FPs, 52 patients (0.3%, 95% CI 0.2–0.4) of all adult presenters in 2018) presented 10 or more times in the 12‐month period from their first presentation in 2018. Among the non‐FPs (1–3 visits in a 12‐month period), 11 761 people presented once (70.7%), 2977 presented two times (17.9%) and 1031 presented three times (6.2%).

### Sociodemographic characteristics of study population

Sociodemographic characteristics are shown in Table 1. Half (51.9%) of the study population were male. The median age was 38 years (interquartile range 28–57) and 16.7% of patients were over age 65. More than half (56.9%) of patients were married and less than 1% (0.9%) of the study population identified as being of Aboriginal or Torres Strait Islander origin.

**TABLE 1:** Sociodemographic characteristics of subjects at index ED presentation, overall and by frequent presenter (FP) classification (non‐frequent, frequent and very frequent), 2018

Sociodemographic characteristic†		*N* (%)		
	All	Non‐frequent ED presenters (1–3 presentations)	Frequent ED presenters (≥4 presentations)	*P*‐value‡
Total	16 624	15 769	855	
Age				
Median (IQR)	38 (28–57)	38 (28–56)	46 (29–72)	<0.001
18–65	13 855 (83.3)	13 263 (84.1)	592 (69.2)	
>65	2769 (16.7)	2506 (15.9)	263 (30.8)	<0.001
Sex				
Male	8626 (51.9)	8222 (52.1)	404 (47.3)	
Female	7998 (48.1)	7547 (47.9)	451 (52.7)	0.005
Marital status, *n* = 16 469				
Married/*de facto*	9372 (56.9)	8917 (57.1)	455 (53.4)	
Other	7097 (43.1)	6700 (42.9)	397 (46.6)	0.03
Country of birth, *n* = 16 583				
Australia	5758 (34.7)	5431 (34.5)	327 (38.2)	
Other	10 825 (65.3)	10 297 (65.5)	528 (61.8)	0.03
Primary language, *n* = 16 590				
English	10 218 (61.6)	9720 (61.8)	498 (58.2)	
Arabic	2033 (12.2)	1870 (11.9)	163 (19.1)	
Mandarin	1180 (7.1)	1152 (7.3)	28 (3.3)	
Turkish	637 (3.8)	596 (3.8)	41 (4.8)	
Other	2522 (15.2)	2397 (15.2)	125 (14.6)	0.04
Aboriginality, *n* = 16 567				
Aboriginal or Torres Strait Islander	152 (0.9)	142 (0.9)	10 (1.2)	
Non‐Aboriginal or Torres Strait Islander	16 415 (99.1)	15 570 (99.1)	845 (98.8)	0.43
Interpreter, *n* = 14 792				
Yes	1964 (13.3)	1847 (13.2)	117 (15.3)	
No	12 828 (86.7)	12 180 (86.8)	648 (84.7)	0.09
Insurance status, *n* = 16 422				
Private insurance	1455 (8.9)	1414 (9.1)	41 (4.8)	
No private insurance	14 967 (91.1)	14 161 (90.9)	806 (95.2)	<0.001

† The number of subjects (*n*) assessed for a characteristic is reported separately if it varies from the study sample total due to missing data.

‡ Statistical tests for null hypothesis of no difference between FP and non‐FP groups: Mann–Whitney *U*‐test for comparison of age distribution, Pearson's *Χ* ^2^ test for categorical variables.

IQR, interquartile range.

One‐third of patients (34.7%) of patients in the study population were born in Australia. The next most frequent countries of birth were Lebanon (10.5%), China (7.5%) and Turkey (4.4%). Most of the study population (61.6%) spoke English at home. Major languages other than English were Arabic (12.2%), Mandarin (4.9%) and Turkish (3.8%).

### *Attendance and clinical characteristics of FPs* versus *non‐FPs*

In the total study population, one‐fifth (20.3%) of patients were brought in by ambulance; most (78.7%) arrived by car or walked in (Table 2). A larger proportion of FPs were brought in by ambulance (30.5%). The majority (81.5%) of patients arrived between 7.00 and 23.00 hours.

**TABLE 2:** Attendance and clinical characteristics for index ED presentation, overall and by frequent presenter (FP) classification (non‐frequent, frequent and very frequent), 2018

Attendance factors†		*N* (%)		
	All	Non‐frequent ED presenters (1–3 presentations)	Frequent ED presenters (≥4 presentations)	*P*‐value‡
Total	16 624	15 769	856	
Referral source to ED, *n* = 16 400				
Self/family/friend	15 530 (94.7)	14 719 (94.6)	811 (95.7)	
GP/dentist/specialist	556 (3.4)	530 (3.4)	26 (3.1)	
Other hospital/health service	119 (0.7)	118 (0.8)	1 (0.1)	
Other	195 (1.2)	186 (1.2)	9 (1.1)	0.13
Mode of arrival, *n* = 16 573				
Ambulance	3365 (20.3)	3102 (19.7)	263 (30.8)	
Car/walked in	13 036 (78.7)	12 447 (79.2)	589 (69.1)	
Other	172 (1.0)	171 (1.1)	1 (0.1)	<0.001
Time of attendance				
07:00–23:00	13 552 (81.5)	12 897 (81.8)	655 (76.6)	
23:01–06:59	3072 (18.5)	2872 (18.2)	200 (23.4)	<0.001
Triage category, *n* = 16 538				
1	55 (0.3)	53 (0.3)	2 (0.2)	
2	2577 (15.6)	2435 (15.5)	142 (16.7)	
3	5454 (33.0)	5113 (32.6)	341 (40.1)	
4	7065 (42.7)	6744 (43.0)	321 (37.8)	
5	1387 (8.4)	1343 (8.6)	44 (5.2)	<0.001
Discharge referral				
GP/LMO/specialist/outpatient	9923 (59.7)	9470 (60.1)	453 (53.0)	
Other health care/community services	5336 (32.1)	4995 (31.7)	341 (39.9)	
Not referred	964 (5.9)	923 (5.9)	41 (4.8)	
Died	3 (0.0)	3 (0.0)	0 (0.0)	
Not known	398 (2.4)	378 (2.4)	20 (2.3)	<0.001
Common presenting problems, *n* = 16 528				
Abdominal pain	2053 (12.4)	1931 (12.3)	122 (14.4)	0.08
Injury	2277 (13.8)	2223 (14.2)	54 (6.4)	<0.001
Chest pain	1342 (8.1)	1259 (8.0)	83 (9.8)	0.07
Care	876 (5.3)	813 (5.2)	63 (7.4)	0.006
Dizziness/syncope	569 (3.4)	541 (3.5)	28 (3.3)	0.81
Headache	444 (2.7)	420 (2.7)	24 (2.8)	0.79
Shortness of breath/asthma	581 (3.5)	518 (3.3)	63 (7.4)	<0.001

† The number of subjects (*n*) assessed for a characteristic is reported separately if it varies from the study sample total due to missing data.

‡ Statistical tests for null hypothesis of no difference between FP and non‐FP groups: Fisher's exact test for categorical variables with subgroup frequency <5, Pearson *Χ* ^2^ test for all other categorical variables. Statistical comparisons between FP and non‐FP groups performed for each common problem individually.

GP, general practitioner.

Common presenting problems differed between non‐FPs and FPs (Table 2). There were more FPs who presented for routine care (*P* = 0.006), for example, for medication administration or shortness of breath or asthma (*P* < 0.001); whereas fewer presented with injury (*P* < 0.001). A small number of primary diagnoses were recorded as mental health; 1.0% in the non‐FPs and 0.5% in the FPs (*P* = 0.19, primary diagnosis recorded for 15 434 [93%] patients). Drug and alcohol‐related primary diagnoses comprised 0.8% of visits in non‐FPs and 0.9% of visits in FPs (*P* = 0.79).

### *Sociodemographic characteristics of FPs* versus *non‐FPs*

Of the sociodemographic characteristics examined for association with FP (Table 3), age >65 *versus* 18–65 years was the strongest determinant (adjusted for sex, OR 2.33; 95% CI 2.01–2.72). Overall, men were less likely to be FPs than women (adjusted for age, OR 0.84; 95% CI 0.74–0.97).

**TABLE 3:** Association between sociodemographic characteristics and frequent presentation, 2018, unadjusted and adjusted odds ratio and 95% confidence interval (CI)

Characteristic	No. FP/*N*	Unadjusted odds ratio (95% CI)	Adjusted odds ratio (95% CI)†
Age in years			
*per year*		1.02 (1.01–1.02)	1.02 (1.01–1.02)
18–65	592/13 855	1.00	1.00
>65	263/2769	2.35 (2.02–2.74)	2.33 (2.01–2.72)
Sex			
Female	451/7998	1.00	1.00
Male	404/8626	0.82 (0.72–0.94)	0.84 (0.74–0.97)
Marital status			
Married/*de facto*	455/9372	1.00	1.00
Other	397/7097	1.16 (1.01–1.33)	1.26 (1.09–1.44)‡
Country of birth			
*All*			
Australia	327/5728	1.00	1.00
Other	528/10 825	0.85 (0.74–0.98)	0.76 (0.66–0.88)
*18–65 years*			
Australia	265/5008	1.00	1.00
Other	327/8810	0.69 (0.58–0.81)	0.67 (0.56–0.79)
*>65 years*			
Australia	62/750	1.00	1.00
Other	201/2015	1.23 (0.91–1.66)	1.36 (1.01–1.84)
Primary language			
*All*			
English	498/10 218	1.00	1.00
Arabic	163/2033	1.70 (1.46–2.04)	1.54 (1.28–1.86)
Mandarin	28/1180	0.47 (0.32–0.70)	0.40 (0.27–0.59)
Turkish	41/637	1.34 (0.97–1.87)	1.08 (0.77–1.51)
Other	125/2522	1.02 (0.83–1.25)	0.91 (0.75–1.12)
*18–65 years*			
English	384/8853	1.00	1.00
Arabic	103/1629	1.49 (1.19–1.86)	1.45 (1.15–1.81)
Mandarin	15/930	0.36 (0.22–0.61)	0.35 (0.21–0.59)
Turkish	22/432	1.18 (0.76–1.84)	1.13 (0.72–1.76)
Other	68/1984	0.78 (0.60–1.02)	0.77 (0.59–1.00)
*>65 years*			
English	114/1365	1.00	1.00
Arabic	60/404	1.91 (1.37–2.68)	2.06 (1.47–2.89)
Mandarin	13/250	0.60 (0.33–1.09)	0.58 (0.32–1.05)
Turkish	19/205	1.12 (0.67–1.87)	1.26 (0.75–2.11)
Other	57/538	1.30 (0.93–1.82)	1.31 (0.94–1.84)
Interpreter required			
*All*			
No	648/12 828	1.00	1.00
Yes	117/1964	1.19 (0.97–1.46)	0.99 (0.80–1.22)
*18–65 years*			
No	478/10 968	1.00	1.00
Yes	51/1392	0.84 (0.62–1.12)	0.81 (0.60–1.09)
*>65 years*			
No	170/1860	1.00	1.00
Yes	66/572	1.30 (0.96–1.75)	1.29 (0.95–1.74)
Private health insurance			
No	806/14 967	1.00	1.00
Yes	41/1455	0.51 (0.37–0.70)	0.52 (0.38–0.72)

† Age adjusted for sex, all other characteristics adjusted for age in years (continuous variable) and sex.

‡ Marital status stratified by sex: males 1.60 (1.30–1.98); females 1.15 (0.95–1.40).

FP, frequent presentation.

Overall, patients born outside Australia were less likely to be FP than Australian‐born patients (adjusted for age and sex, OR 0.76; 95% CI 0.66–0.88). This association varied by age (interaction *P* < 0.001). Thus, we also assessed the association separately by age group. In 18‐ to 65‐year old, those born outside Australia were less likely to be FPs (OR 0.67; 95% CI 0.56–0.79, *P* < 0.001). Conversely, in those over age 65, FPs were less likely to be Australian born (OR 1.36; 95% CI 1.01–1.84).

FP was more likely for Arabic speakers compared to English speakers (adjusted OR 1.54; 95% CI 1.28–1.86), and least likely for Mandarin speakers (adjusted OR 0.40; 95% CI 0.27–0.59). These associations were consistently observed in each age group (Table 3).

Overall, we did not observe an association between requiring an interpreter and FP (adjusted OR 0.99; 95% CI 0.80–1.22). This association varied by age (interaction *P* < 0.001). Those in the >65 age group were more likely to be FPs if they required an interpreter (adjusted OR 1.29; 95% CI 0.95–1.74, *P* = 0.10). In the 18–65 age group, those requiring an interpreter were less likely to be FP (adjusted OR 0.81; 95% CI 0.60–1.09, *P* = 0.17). Both results were not statistically significant. Those with private health insurance were less likely to be FPs (OR 0.51; 95% CI 0.37–0.70, *P* < 0.001).

## Discussion

Our finding that FPs represent 5.1% of total patients presenting to Auburn Hospital is consistent with the range reported in Australia and internationally.^1^, ^10^ FP visits represented 15.8% of the total ED visits, which reinforces that FPs represent a small proportion of ED patients, they place a significant burden on ED resources. Furthermore, it demonstrates that FPs represent a significant burden for the health system at non‐referral hospitals. This is consistent with evidence for larger referral hospitals'.^8^, ^9^

As previously mentioned, Auburn Hospital ED is situated in one of the most CALD areas in NSW. Therefore, country of birth and language spoken at home were important variables to explore. We found that patients over 65 who were born outside of Australia are more likely to be FPs, which is consistent with other studies.^1^, ^3^, ^7^, ^8^, ^9^ Conversely, an inverse relationship was found with patients aged 18–65 born outside Australia.

We also found that Arabic speakers (who comprised 13% of the study population, consistent with the Auburn population proportion)^15^ were most likely to be FPs, whereas Mandarin speakers (who comprised 7% of the study population, lower than the Auburn population proportion) were least likely. Although migrants generally experience lower mortality than the Australian‐born population across all age groups,^16^ the pattern found in our study highlights the importance of disaggregating language and cultural groups to understand the specific needs of different groups and which groups may be most at risk of FP in Australia. In particular, although literacy in English was not measured and may vary between primary language groups, our finding of lower ED attendance among Mandarin speakers than English speakers suggests the differences observed in ED attendance between language groups points to broader socio‐economic‐cultural differences than English language skills alone. We propose that health literacy may be an important factor mediating the association between older age and FP, as well as health disparities between CALD and non‐CALD populations and unique challenges such as language barriers and discrimination within the system.^11^ Health literacy is defined as, ‘the ability of people to access, understand and apply information about health and the healthcare system so as to make decisions that relate to their health’.^17^ It may be significantly lower in CALD populations,^18^ which results in decreased engagement with health services and decreased ability to self‐manage. Health and illness are also concepts shaped by cultural values and beliefs. These may also influence preferences and priorities as well as health‐seeking behaviour.^19^ Further research is needed to explore the association between health literacy, culture and FPs. To date, there is limited evidence about the effectiveness of interventions that aim to reduce ED use by CALD populations.^20^, ^21^

Our results show that age over 65 was the most significant sociodemographic determinant of FP. Other studies have similarly found increased rates of FP in the elderly.^5^, ^7^, ^22^ Although older patients are more likely to have chronic diseases requiring ongoing care,^17^, ^22^ this highlights the need for further research and interventions to improve care coordination between health services and aged care services.^5^ Previous literature has shown evidence for case management^20^ and enhanced geriatric outreach services such as Hospital in the Nursing Home.^18^

Our study found that private health insurance reduced the risk of FP. This reinforces previous findings, and may highlight a link between socioeconomic status (SES) and FP. Although our data set did not provide variables to estimate SES directly, health insurance status may function as a proxy measure. The Australian Bureau of Statistics reports low rates of private health insurance for people who are unemployed and living in areas of high socioeconomic disadvantage.^23^ This hypothesis is reinforced by previous research, which has highlighted the association between low SES and frequent presentation to the ED.^18^ The Auburn Hospital catchment comprises an area of low SES, with a median weekly personal income of A$434, lower than the NSW average of A$664 and a higher unemployment rate than the rest of NSW.^15^ Given the protective nature of private health insurance, our results further suggest that low SES is associated with FP. This may be an important predictor to look at in future studies on FPs.

Finally, several Australian studies have previously shown that mental health issues, drug and alcohol use and urgency of presentation are predictive of FP.^7^, ^8^, ^10^, ^24^, ^25^ Similarly, in the international FP literature, substance abuse and mental health or behavioural health problems are consistent and significant predictors.^3^, ^4^ Our study, in contrast, did not find a statically significant association between alcohol and drug use and FP, or mental health and FP. However, there were relatively few ED presentations with a primary diagnosis of these conditions which limited our ability to investigate these associations. Auburn is not a gazetted mental health facility and thus is an ambulance bypass for those who are scheduled requiring mental health assessment, and the hospital does not have drug and alcohol cover.

### Strengths and limitations

This is a single‐centre study of a hospital in a CALD area. However, it offers unique insights into the characteristics of FPs at Auburn ED. It highlights important characteristics to consider of FPs in Australia. Future research could expand to hospitals with similar CALD populations. As well as explore if there is access to culturally appropriate primary care services and primary care providers to avoid routine care presentations to ED in these areas. However, it is important to highlight that research has shown that the use of EDs to seek routine care is a common occurrence.^26^

The limitations of the database, including lack of ICD10AM codes for clinical diagnoses, were the major disadvantages. Incomplete data on the use of an interpreter and its association with frequent presentation limits the use of this information to inform service design. Furthermore, data on presenting complaints and ED clinical diagnoses, including drug and alcohol use and mental health, were missing for many patients, which limited our ability to draw conclusions about the clinical characteristics of FPs.

## Conclusion

Frequent attendance to the ED places a significant burden on the resources of the health system. Many FPs have unique needs that should be addressed within and outside of the hospital. This study found that the proportion of FPs at Auburn was consistent with the range reported in Australia and internationally. The association between age and FP was also consistent with previous research. Older patients born outside Australia and Arabic speakers were more commonly FPs. Identifying ways to provide targeted services to those in the >65 age group who frequently present to the Auburn ED may reduce the overall rates of FP. In addition, further research into language and ethnicity‐related rates of FP is required to provide appropriate care to this patient group.

## Data Availability

The data that support the findings of this study are available on request from the corresponding author. The data are not publicly available due to privacy or ethical restrictions.
